# Determination of natural antibodies, beta-hydroxybutyric acid, and non-esterified fatty acid levels in the serum of peripartum Tuj and Hemşin sheep

**DOI:** 10.14202/vetworld.2021.1002-1006

**Published:** 2021-04-26

**Authors:** Cihan Kaçar, Semra Kaya, Mushap Kuru, Ekin Emre Erkılıç, Metin Öğün, Hasan Oral, Murat Can Demir

**Affiliations:** 1 Department of Obstetrics and Gynecology, Faculty of Veterinary Medicine, Kafkas University, 36100 Kars, Turkey; 2 Department of Internal Medicine, Faculty of Veterinary Medicine, Kafkas University, 36100 Kars, Turkey; 3 Department of Biochemistry, Faculty of Medicine, Kafkas University, 36100 Kars, Turkey

**Keywords:** beta-hydroxybutyric acid, ewe, natural antibody, non-esterified fatty acid, peripartum period

## Abstract

**Background and Aim:**

Many metabolic and immunological changes occur during the transition period. Innate immunity plays an important role against to infections and natural antibodies (NAb) are important in immunity. This study aims to determine a connection between serum NAb titers, beta-hydroxybutyric acid (BHBA), and non-esterified fatty acid (NEFA) concentrations in Tuj and Hemşin sheep during the peripartum period.

**Materials and Methods:**

Serum NAb, BHBA, and NEFA levels were determined from the blood samples collected from Tuj and Hemşin sheep on days 30 and 15 before birth, on the day of birth (day 0), and on days 15 and 30 after birth.

**Results:**

NAb titers were found to be higher in Tuj than in Hemşin sheep (p<0.001). No statistically significant difference was found in serum BHBA concentrations of both breeds on all sampling days (p>0.05). The serum NEFA level was lower in Tuj sheep in the last 15 days of pregnancy compared to Hemşin sheep (p<0.05), while no difference was found in samples collected at the other time points.

**Conclusion:**

This study indicated that serum NAb titers significantly changed in Tuj and Hemşin sheep during the transition period. Serum BHBA and NEFA concentrations increased during the last stages of pregnancy and decreased after birth. Based on these findings, it is suggested that the immunological status could vary by the breed of sheep or various factors that affect the sheep’s metabolic state.

## Introduction

In mammals, innate immunity plays an important role in preventing and treating infections [1]. Natural antibodies (NAb) are important in humoral immunity [2]. They are defined as antigen-binding and considered a humoral part of the innate immune system in non-immunized individuals [3]. NAb originate from CD5+ and B1 cells in mammals [4] and are mostly the isotopes of immunoglobulin (Ig) M [5]. In cooperation with the complement system, they serve as the first defense mechanism of the immune system [6]. They bind directly to pathogens [7,8], contributing to their direct neutralization, and play a role in the defense mechanism by the activation of the complement system [9].

In sheep, energy requirements increase with the rapid growth of the fetus in the last stages of pregnancy. Insulin and prolactin affect carbohydrate and lipid metabolism and can pose a risk of pregnancy toxemia [10,11], which occurs as a result of the negative energy balance due to the increased energy requirements of the fetus [12,13]. The hormonal effects in the late stages of pregnancy lead to decreased levels of glucose and increased fatty acid and ketone body concentrations in carbohydrate and fat metabolism [14,15]. The main indicators of sheep’s energy status are blood beta-hydroxybutyric acid (BHBA) and non-esterified fatty acid (NEFA) concentrations in the peripartum period [16,17]. In addition, serum NEFA concentration reflects fat mobilization, and BHBA indicates fat oxidation in the liver [18,19]. Limited information exists on the presence and functions of NAb in sheep in the peripartum period.

This study aimed to investigate the link between serum NAb titers and the BHBA and NEFA concentrations in Tuj and Hemşin sheep during the peripartum period.

## Materials and Methods

### Ethical approval

This study was conducted after obtaining approval from the Kafkas University Animal Experiments Local Ethics Committee (KAU-HADYEK-2015-016).

### Study period and location

The study was conducted from December-2015 to April-2016 at the Kafkas University, Veterinary Faculty, Research and Application Farm in Kars province, which is located at 40.5^°^ north latitude and 43^°^ east longitude.

### Animals

The present study was carried out with Tuj (n=25) and Hemşin (n=25) sheep housed under similar care and feeding conditions.The animals were fed with hay and concentrate (12% crude protein, 2600 kcal/kg) twice a day and water *ad libitum*. The ewes were naturally mated, and pregnancies were determined by ultrasonography on day 30. In the past 30 and 15 days of pregnancy, blood was collected from the vena jugularis into gel serum vacuum tubes (8.5 mL) to obtain the serum. Blood was also collected on the day of delivery (day 0), and on days 15 and 30 postpartum. The blood samples were centrifuged at 3000 rpm (1600 g) for 15 min. Then, the serum was transferred to Eppendorf tubes and stored at -20°C until the analyses. Body condition scores of sheep were evaluated according to a 5-point scale from 1=weak to 5=obese [20].

### NAb, BHBA, and NEFA analysis

In the present study, the NAb titers connected to keyhole limpet hemocyanin (KLH) were determined based on the indirect enzyme-linked immunosorbent assay (ELISA) method [21]. The plates were coated with 2 mg KLH/mL dissolved in carbonate buffer (10.6 g/L Na_2_CO_3_, and pH 9.6). After incubating at 4°C overnight, the plates were washed once with tap water and 0.05% Tween 20, and then 2.5% rabbit serum was added. The plates were incubated with 0.05% Tween 20 in phosphate-buffered saline at room temperature for 1 h; then, 1:4 diluted serum samples and 2.5% rabbit serum were added to the wells. After washing with tap water and 0.05% Tween 20, 100 mL of 1:20,000-diluted peroxidase-bound rabbit-anti-sheep-Ig G was added to each well. After washing again, 100 mL of tetramethylbenzidine was added to each well, and the wells were incubated for 10 min at room temperature. The reaction was stopped by adding 50 mL of 1.25 M H_2_SO_4_ to each well, and the results were determined at 450 nm with an ELISA Reader (Epoch^®^, BioTek, USA).

BHBA (Ranbut^®^, Randox, UK) and NEFA (Wako Diagnostics, VA) levels in the sera were determined by the Spectrophotometric measurement (Epoch^®^, Biotek, USA)method using commercial kits at Kafkas University, Faculty of Veterinary Medicine, Department of Biochemistry.

### Statistical analysis

Statistical analyses were performed using the SPSS^®^ (SPSS 18.0, IL, USA) software program. When the normality assumptions were made for the serum NAb titer, NEFA, and BHBA concentrations, statistical differences were detected among sampling days (−30, −15, 0, 15, and 30) by applying repeated analysis of variance and Bonferroni tests. The differences in NAb titer, NEFA, BHBA concentrations and the body condition scores due to sheep breed were demonstrated by independent t-tests. Results are presented as mean±standard error (X±SE). Values with p<0.05 were considered statistically significant.

## Results

In the present study, initial body condition scores were determined to be 3.44±0.08 in Tuj sheep and 3.42±0.07 in Hemşin sheep and were similar in every period analyzed without any significant difference (p>0.05). Twin births were observed in 1 Tuj and 3 Hemşin ewes. Birth of triplets or more did not occur. Since the number of multiple births was low, litter size was not used as a factor in the model.

**Figure-1:**
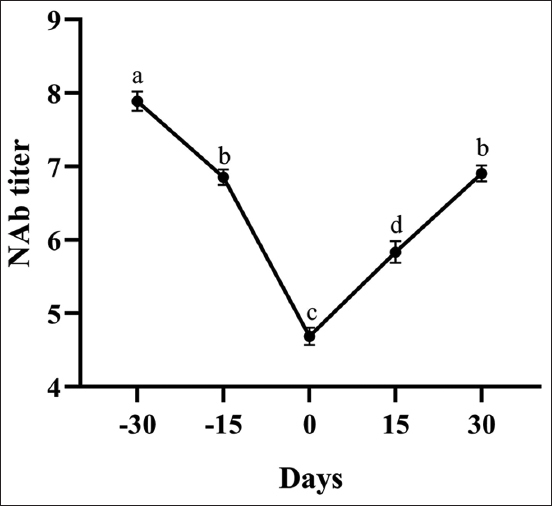
Serum natural antibody titers in all sheep.

**Figure-2:**
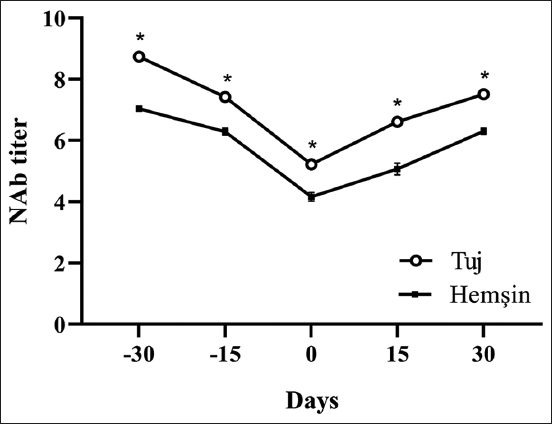
Serum natural antibody titer bound to keyhole limpet hemocyanin in Tuj and Hemşin sheep *p<0.001.

Natural antibody titers decreased in all sheep during the past 30 days of pregnancy and then increased in 30 days after birth. Serum NAb titers were detected to be at similar levels in the past 15 days of pregnancy and 30 days after birth; however, a statistical difference was found among the levels of serum NAb titers on the other days of the study (p<0.001; Figure-1). Figure-2 shows the NAb titers binding to KLH in the groups. The NAb titers were higher in Tuj sheep compared to Hemşin sheep during the whole experimental period (p<0.001).

In all sheep, both BHBA and NEFA levels increased during the past 30 days of pregnancy and decreased after birth. The reduction or increase in NEFA concentrations were found to be statistically significant (p<0.001). Serum BHBA concentration was found to be statistically significantly higher on prepartum 15 and postpartum 15 days (p<0.01) and the other application days (p<0.001). Table-1 shows the serum BHBA and NEFA concentrations.

**Table 1:** Serum BHBA and NEFA concentrations in prepartum and postpartum periods in all sheep (n=50).

Parameters	Days					p-value

	−30	−15	0 (Parturition)	15	30	
BHBA (mmol/L)	0.44±0.01^a^	0.56±0.01^b^	0.72±0.01^c^	0.59±0.01^d^	0.49±0.01^e^	<0.001
NEFA (mmol/L)	0.43±0.01^a^	0.55±0.01^b^	0.71±0.01^c^	0.59±0.01^d^	0.48±0.01^e^	<0.001

^a-e^Difference between values in the same row with different letters is significant. BHBA=Beta-hydroxybutyric acid, NEFA=Non-esterified fatty acid

No statistical difference was detected in serum BHBA concentrations between Tuj and Hemşin breeds in any of the periods evaluated (p>0.05). The serum NEFA level was determined to be lower in Tuj sheep compared to Hemşin sheep during the past 15 days of pregnancy (p<0.05), with no significant difference found on other days. Table-2 shows the serum NAb titer and BHBA and NEFA concentrations.

**Table 2:** Serum NAb titers and BHBA and NEFA concentrations in Tuj (n=25) and Hemşin (n=25) sheep during prepartum and postpartum periods.

Parameters	Breed	Days					p-value

		−30	−15	0 (Parturition)	15	30	
BHBA (mmol/L)	Tuj	0.44±0.01^A^	0.55±0.01b^B^	0.71±0.01^C^	0.59±0.01^B^	0.49±0.01^D^	<0.001
	Hemşin	0.43±0.01^A^	0.56±0.01^B^	0.72±0.01^C^	0.59±0.01^B^	0.49±0.01^D^	<0.001
NEFA (mmol/L)	Tuj	0.42±0.01^A^	0.53±0.01^aB^	0.69±0.01^C^	0.60±0.01^D^	0.49±0.01^B^	<0.001
	Hemşin	0.43±0.01^A^	0.56±0.01^bB^	0.73±0.02^C^	0.59±0.01^B^	0.48±0.01^D^	<0.001
NAb titers	Tuj	8.74±0.06^aA^	7.42±0.07^aB^	5.22±0.11^aC^	6.61±0.07^aD^	7.51±0.09^aB^	<0.001
	Hemşin	7.04±0.08^bA^	6.29±0.11^bB^	4.16±0.14^bC^	5.07±0.19^bD^	6.30±0.10^bB^	<0.001

^A-D^Difference between values in the same row with different letters is significant. ^a: b^The difference between values with different letters in the same column is significant, p*<*0.05. NAb=Natural antibody, BHBA=Beta-hydroxybutyric acid, NEFA=Non-esterified fatty acid

## Discussion

Limited information exists on the alteration in the immune system of sheep during the peripartum period. To date, no studies have been conducted on the levels of NAb in sheep during pregnancy and the postpartum period. Many physical and pathogenic stress factors are reported to affect antibody release [22]. This study evaluated the relationship between the serum NAb titers and the BHBA and NEFA concentrations in the prepartum and postpartum periods in Tuj and Hemşin sheep. While no previous studies exist on NAb titers in sheep, it is known that metabolic and stress factors lead to changes in the immune system in cows during the transition from pregnancy to lactation [23,24]. It is reported that the NAb concentration in blood and milk during the early lactation period reflects the metabolic health status and that the NAb concentration and activation increases in pregnant cows [3]. In the present study, the serum NAb titer was found to decrease during the past 30 days of pregnancy and to increase in the postnatal period. The decrease in the serum NAb level is attributed to the increase in physical and metabolic stress due to fetal growth in the last period of pregnancy. The most important findings of this study were that serum NAb concentrations were higher in Tuj sheep than in Hemşin sheep during the whole experimental period, and that Tuj sheep were more resistant to environmental and nutritional conditions compared to Hemşin sheep.

Metabolic diseases in ruminants, such as fatty liver and ketosis, occur due to the inability to adapt to a negative energy balance [25]. The amount of NEFA increases as a result of fatty liver; and as a result of oxidation of hepatic fatty acids, it becomes esterified in triglyceride. Fatty liver causes an increase in the risk for ketosis, abomasum displacement, metritis, and immunosuppression and a decrease in reproductive efficiency [26]. A negative energy balance has harmful effects on sheep and causes immunosuppression. Immunosuppression, in turn, leads to an increase in infectious disease incidents such as metritis [27]. As a result of negative energy balance, blood NEFA and glycerol are mobilized from body fat for energy production. Most of the NEFAs are used in the synthesis of ketone bodies. Tracking the serum BHBA concentrations is useful for monitoring the energy status in pregnant sheep [28].

A correlation was already indicated between the NAb binding protein, KLH, and body condition score, energy balance, milk fat, and plasma cholesterol concentration [3]. It has been reported that serum BHBA concentrations do not vary in sheep with different body condition scores [16]; however, both the NEFA and BHBA concentrations were found to be higher in the prepartum period than in the postpartum period in Lori-Bakhtiari sheep, and significant changes were reported in blood NEFA and BHBA levels during the peripartum period [29]. Serum BHBA concentration was reported to be significantly higher in sheep in the dry period compared to the lactation period [30]. In sheep, during the peripartum period, the serum BHBA concentration increased when the body condition score was below 2.75 or above 3.5 [31]. It was found that the mean BHBA and NEFA concentrations were higher before birth compared to the postnatal values [31]. A high NEFA concentration in animals that are not prone to any disease indicates a negative energy balance [28]. It has been reported that the increase in serum NEFA and BHBA in the near-term period may be related to the decrease in feed intake due to stress and hormonal changes during lambing [29]. In the present study, a negative relationship was found between the serum NAb and NEFA concentrations. This finding proves that there is a close relationship between healthy metabolism and NAb [3]. In the present study, in parallel with the previous studies, the serum NEFA and BHBA levels were observed to increase above the normal values from the 30^th^ day of pregnancy to delivery and to decrease after birth. It was observed that both serum BHBA and NEFA concentrations decreased by the 30^th^ day after birth. A statistically significant difference was determined between the NEFA concentrations in the sheep during the experiment. This is attributed to the suppression of the stress factors by the immune system as the pregnancy progresses and the energy deficiency that develops in parallel with the growing offspring. On the 15^th^ day before birth, the serum NEFA level was found to be significantly lower in Tuj sheep compared to Hemşin sheep. The serum NAb titer decreased while serum BHBA and NEFA concentrations increased in sheep in the prenatal period.

## Conclusion

The serum NAb titer significantly changed during the transition period in Tuj and Hemşin sheep. The serum BHBA and NEFA concentrations increased during the last stages of pregnancy and started to decrease after birth. These findings suggest that the immunological status may vary by the breed and the metabolic state of ewes.

## Authors’ Contributions

SK, CK, and MK: Designed the experimental procedures. SK, CK, MK, HO, and EEE: Conducted the research work. MÖ: Helped in biochemical analysis. CK and MCD: Performed statistical analysis. CK, MK, MÖ, and MCD: Prepared figures, tables, revised, and submitted the manuscript. All authors read and approved the final manuscript.
